# Genomic-driven nutritional interventions for radiotherapy-resistant rectal cancer patient

**DOI:** 10.1038/s41598-023-41833-8

**Published:** 2023-09-08

**Authors:** Joshua Southern, Guadalupe Gonzalez, Pia Borgas, Liam Poynter, Ivan Laponogov, Yoyo Zhong, Reza Mirnezami, Dennis Veselkov, Michael Bronstein, Kirill Veselkov

**Affiliations:** 1 Department of Computing, Imperial College London, London, SW7 2BX UK; 2 Prescient Design, Genentech, Basel, 4052 Switzerland; 3 North Middlesex University Hospital, London, N18 1QX UK; 4 Department of Surgery and Cancer, Imperial College London, London, SW7 2BX UK; 5 Royal Free Hospital, London, NW3 2QG UK; 6 Department of Computer Science, University of Oxford, Oxford, OX1 3QD UK; 7 Department of Environmental Health Sciences, Yale University, New Haven, CT 06510 USA

**Keywords:** Cancer, Computational biology and bioinformatics, Drug discovery, Systems biology

## Abstract

Radiotherapy response of rectal cancer patients is dependent on a myriad of molecular mechanisms including response to stress, cell death, and cell metabolism. Modulation of lipid metabolism emerges as a unique strategy to improve radiotherapy outcomes due to its accessibility by bioactive molecules within foods. Even though a few radioresponse modulators have been identified using experimental techniques, trying to experimentally identify all potential modulators is intractable. Here we introduce a machine learning (ML) approach to interrogate the space of bioactive molecules within food for potential modulators of radiotherapy response and provide phytochemically-enriched recipes that encapsulate the benefits of discovered radiotherapy modulators. Potential radioresponse modulators were identified using a genomic-driven network ML approach, metric learning and domain knowledge. Then, recipes from the Recipe1M database were optimized to provide ingredient substitutions maximizing the number of predicted modulators whilst preserving the recipe’s culinary attributes. This work provides a pipeline for the design of genomic-driven nutritional interventions to improve outcomes of rectal cancer patients undergoing radiotherapy.

## Introduction

Mesorectal excision is the surgical standard of care in rectal cancer (RC)^1^. The additive benefit of radiotherapy (RT) in reducing local recurrence in advanced RC has been extensively documented^2–5^. However, there is considerable variability in radioresponse across patients, with patients showing either (1) complete tumor destruction, (2) moderate tumor regression, or (3) negligible tumor shrinkage. For patients in the last category, the delay in proceeding to tumor excision while completing RT may increase the likelihood of distant metastases, therefore, modulation of radioresponse to improve RT outcomes is a critical need.

**Figure 1:**
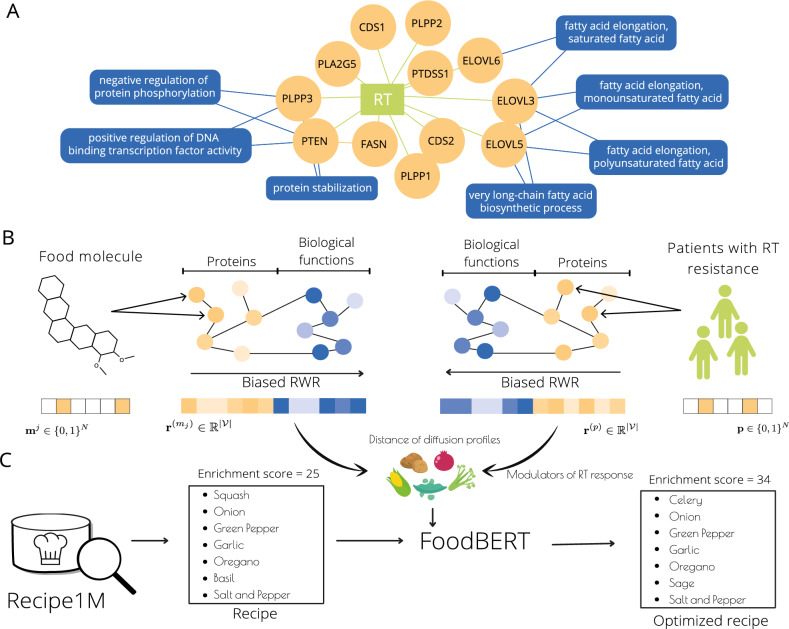
Overview of approach. (**A**) Network representation of over-expressed proteins (yellow) and biological functions (blue) in RT-resistant RC patients. Over-expressed proteins were experimentally identified by one of the authors of this work. Their corresponding biological functions were extracted from the Gene Ontology’s Biological Processes^32^. Over-expression of all genes but PTEN leads to increased lipid availability resulting in cancer-promoting effects including increased nutrient storage (CDS1, CDS2), activation of stress and response signaling pathways (PLA2G5, ELOVL family, FASN and PLP family), and increased immunosuppressive properties (PTDSS1). (**B**) Radioresponse modulators identification module. Food protein targets and RT-resistant-associated proteins are mapped onto a multiscale interactome of proteins and biological functions. A biased random walk with restarts (RWR) propagates the effects of food molecules and phenotype, revealing the most affected proteins and biological functions. Top food molecules with the most similar propagated profiles to the phenotype are used to create a list of food ingredients with potentially beneficial RT response modulation activity. (**C**) Recipe generation module. Using FoodBERT, Recipe1M recipes are optimized to increase the number of ingredients with beneficial radioresponse modulation properties.

RT response is governed by various molecular mechanisms including response to stress, cell death, and cell metabolism^6^. Current strategies to improve radioresponse focus on the modulation of cell death and response to stress using chemotherapies, such as fluorouracil (5-FU), capecitabine, gemcitabine, and cisplatin, to enhance tumor sensitivity to RT^7^. However, combined therapy often produces mixed results and can increase toxicity in normal tissues^7^. In contrast, bioactive molecules within foods appear as a promising alternative to modulate radioresponse, through lipid metabolism modulation^8,9^. Proteins corresponding to up-regulated genes in RT-resistant RC patients participate in lipid biosynthetic and metabolic pathways with various roles (Fig. 1A). The up-regulation of most of these genes translates into increased lipid availability, which leads to a myriad of downstream tumor-promoting effects. For example, CDS1- and CDS2-encoded proteins regulate growth and maturation of lipid droplets which serve as storage, providing nutrients necessary for cell growth, and can serve as additional nutrients for the uncontrolled growth of cancerous cells^10^. Moreover, over-expression of PLA2G5, the ELOVL family of genes, FASN and the PLP family of genes translates into increased lipid availability leading to downstream activation of inflammation and stress pathways. Proteins encoded by these genes increase lipid availability through different mechanisms: PLA2G5-encoded protein through the generation of lysophospholipids and free fatty acids, including arachidonic acid^10,11^; encoded proteins by the ELOVL family of genes through the elongation of long chain fatty acids to provide precursors for synthesis of sphingolipids and ceramides^10,12^; FASN-encoded protein through the synthesis of long-chain fatty acids^10^; and encoded proteins by the PLP family of genes through the hydrolysis and uptake of lipids from extracellular space^10,13^. Increased lipid availability in cancer cells can also lead to increased immunosuppressive properties, as is the case with PTDSS1 over-expression, whose encoded protein catalyzes the formation of phosphatidylserine which, exposed on the surface of tumor cells, increases their immunosuppressive properties and facilitates tumor growth and metastasis^10,14^. On the other hand, PTEN has documented tumor-suppressing properties^10^. Loss of PTEN leads to elevated de novo lipogenesis through induction of SREBP and FASN expression^15^. Therefore, over-expression of PTEN in this context might be a compensatory mechanism to inhibit FASN in an attempt to decrease lipogenesis.

Bioactive molecules in food can modulate lipid metabolism, have a promising safety profile in toxicity studies, and have documented chemopreventive and chemotherapeutic effects^16–18^. This means dietary interventions could be a promising strategy to increase treatment efficacy, prevent resistance acquisition and reduce side effects^19^. However, experimental large-scale testing of chemotherapeutic or chemopreventive properties of bioactive molecules within food is not generally feasible due to a large number of food-based bioactive molecules. As a result, a unique wave of research has leveraged network machine learning (ML) and genomic data to carry out a large-scale screening of anticancer molecules within food^20–22^. Building on these works, we propose a computational genomic-driven approach to mine the space of bioactive molecules within food for potential radioresponse modulators and propose phytochemically-enriched recipes to improve radioresponse of RC patients.

The proposed pipeline, shown in Fig. 1, comprises (1) Identifying over-expressed proteins in RT-resistant RC patients (Fig. 1A) (2) a radioresponse modulators identification module (Fig. 1B) and (3) a recipe generation module (Fig. 1C). In order to identify radioresponse modulators, we map food molecule protein-coding gene targets and RT resistance dysregulated genes onto a heterogeneous network representing proteins and biological functions. Using a network propagation algorithm combined with metric learning, we learn effects of food molecules and the phenotype across the heterogeneous network, and find food molecules with similar effects to those observed in the phenotype. The third stage involves recipe optimization to maximise the number of ingredients with these molecules. Dietary recommendations can then be proposed for RT-resistant RC patients using these recipes and taking into account other user-specific requirements such as taste preferences and allergies.

## Results

### Random walks and metric learning to predict drug-phenotype associations

**Figure 2:**
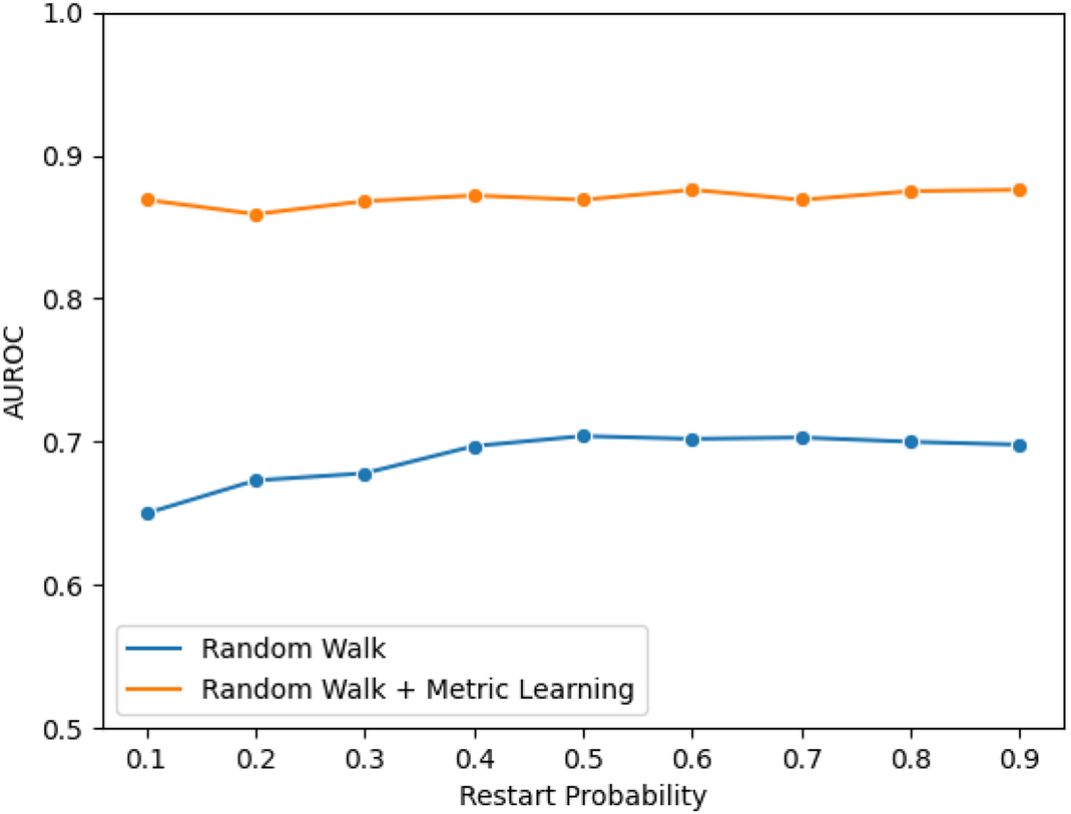
The presented model outperforms the baseline approach across all values of restart probability. AUROC of purely random walk based approach^23^ and the proposed approach combining the method with metric learning as a function of restart probability on the drug-disease prediction task.

We compute propagated profiles of drugs and diseases on the multiscale-interactome using random-walk with restarts and then use metric learning to minimise the distance between a disease and drugs that treat this disease and maximise the distance between a disease and drugs with no known benefit. To show the gain of combining metric learning with the random walk algorithm, we evaluated the improvement on the multiscale-based drug-disease prediction task proposed in^23^. We show that the addition of metric learning improves the random walk diffusion profiles resulting in a 20% increase in performance ($AUROC = 0.714\ vs\ 0.876$). Additionally, the choice of the restart probability only has a small effect on the results in the initial implementation and no influence when combined with metric learning. These results, shown in Fig. 2, confirm the benefit of fixing the restart probability and instead of optimising the weights of the walker, optimizing an MLP by directly back-propagating information from the prediction task using a triplet loss function.

### The model identifies molecules with therapeutic potential to reverse RT resistance

**Figure 3:**
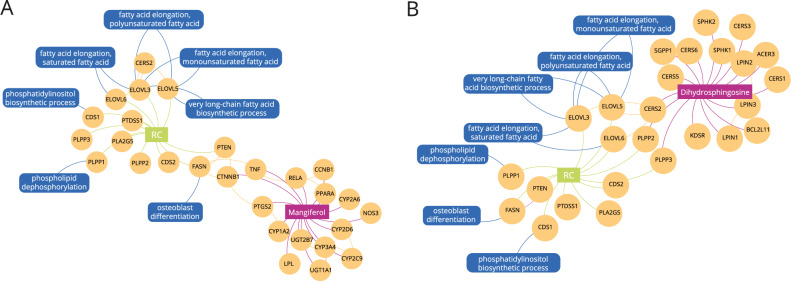
The multiscale interactome identifies proteins and biological functions related to RT modulation. (**A**) The multiscale interactome involving Mangiferol and RT modulation. Mangiferol modulates RT response by targeting CTNNB1 and TNF which has downstream effects in osteoblast differentiation by being linked to FASN. (**B**) The multiscale interactome involving Dihydrosphingosine and RT modulation. Dihydrosphingosine modulates RT response primarily by targeting PLPP2, PLPP3 and CERS2, which are linked to the ELOVL family of genes, inhibiting fatty acid biosynthetic and elongation processes.

Using propagated profiles, we find the top 100 food molecules closest to the phenotype. These molecules affect similar proteins and biological functions as those responsible for radioresistance, however, diffusion profiles do not provide information whether the modulation is positive or negative. Experimental evidence indicates that the phenotype-associated genes are over-expressed in patients exhibiting RT resistance leading to a positive modulation of lipid metabolism (Fig. 1A). Therefore, we use domain knowledge and literature search to filter out identified molecules with positive regulatory effects on lipid metabolism, leaving 33 modulators to retrieve the list of ingredients (Appendix A). Modulators belong to a myriad of compound classes including flavonoids, isoflavonoids, and bezenoids, in alignment with the current knowledge on chemotherapeutic bioactive molecules within foods^11^. Overall, predicted modulators are involved in cell signaling, cell growth and lipid metabolism. For example, Mangiferol and Dihydrosphingosine modulate downstream effects linked to fatty acid biosynthetic and elongation pathways, down-regulating stress and inflammation processes (Figure 3). Additionally, we have compiled a list of ingredients with the highest number of modulators (Appendix B).

### Highest scoring foods modulating RT response

**Figure 4:**
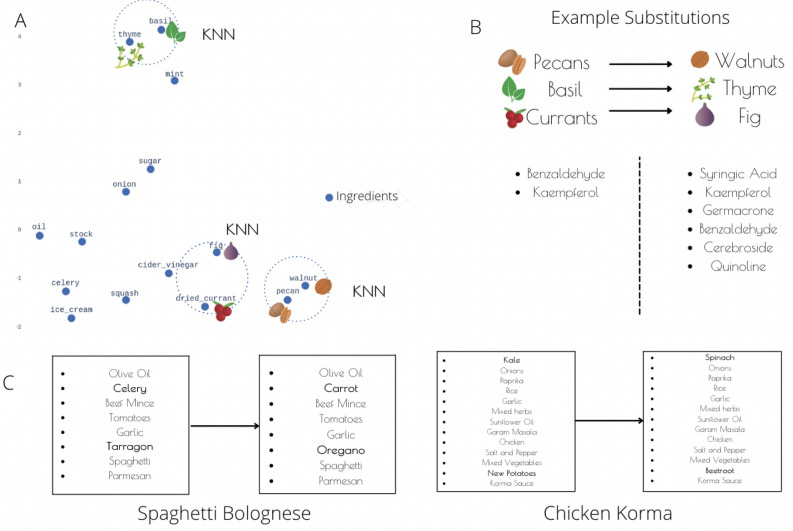
Ingredient substitutions using FoodBert embeddings and food-chemical information. (**A**) Visualisation of FoodBert embeddings. 2D representations found using PCA of the 768 dimensional FoodBert embeddings for some ingredients. Ingredients close in this space appear in similar contexts. (**B**) Example ingredient substitutions. Some example substitutions found by the K-nearest neighbor algorithm in the embedding space and additional filtering to increase the number of beneficial bioactive molecules. (**C**) Some example ingredient substitutions within popular recipes.

In order to validate the recipes, we explored the mechanisms by which the substituted ingredients could modulate radiotherapy response. The tables in appendices A and B give a more extensive treatment of the RT response modulators within food and their potential mechanism for modulation. In Fig. 4, we show how a chicken korma recipe is mutated by substituting kale for spinach and new potatoes for beetroot. Whilst it is difficult to evaluate these substitutions from a culinary perspective, the substitutions do increase the number of potential radioresponse modulators. New potatoes contain none of the found potential modulators, whereas beetroot contains both kaempferol and syringic acid. It has been shown that syringic acid-treated cells developed anti-cancer activities by losing MMP, cell viability, and enhancing intracellular ROS and kaempferol has been shown to be a potential chemo-therapeutic agent to be used alone or in combination with 5-FU to overcome colon cancer drug resistance^24^^25^. Additionally, spinach also contains kaempferol as well as alpha-lipoic acid. Alpha-lipoic acid can effectively induce apoptosis in human colon cancer cells by a mechanism that is initiated by an increased uptake of oxidizable substrates into mitochondria^26^. The addition of these molecules in the recipe, which have been found using our drug-disease association model, and have demonstrated chemotherapeutic effects could be beneficial to radiotherapy-resistant rectal cancer patient as an added measure alongside their standard treatment.

## Discussion

In 2017, dietary risk factors were attributed to approximately 11 million deaths globally, equivalent to about 1 in 5 deaths^27^. This stark statistic emphasises the global need for dietary improvements. Furthermore, evidence has mounted on the potential benefits of drug-like molecules in foods against diseases such as cancer^28,29^, Covid-19^30^ and other health conditions^31^. The prospect of dietary recommendations both for the general population and patients with specific diseases becomes increasingly important. We delved into understanding the role of bioactive food molecules as potential modulators of radiotherapy response. This was achieved by expanding a drug-disease prediction model based on RWR with metric learning, pinpointing radioresponse modulators and showcasing enhanced results on a benchmark dataset. The integration of these analytical methodologies is pivotal; it not only facilitates a comprehensive understanding of intricate interactions but also combines the strengths of prediction and metric learning, ensuring a system-wide appraisal of the potential therapeutic influence of bioactive food molecules on radiotherapy efficacy. By utilising propagated profiles from our model, we identified radioresponse modulators in food, subsequently integrating this with experimental evidence from literature reviews to determine the modulation direction - either positive or negative. It is important to acknowledge, however, that while our findings are encouraging, the model’s transfer-ability may necessitate further validations. This arises from discrepancies, albeit reasonable, in data distribution between the dataset for optimisation of propagation weights and the datasets for food molecules and phenotypes (Appendix C). In this study, we adopted an assumption of direct correspondence between effects on genes and proteins, neglecting potential post-translational modifications. These modifications could be profoundly influenced by dietary intake and merit further exploration in subsequent studies. In terms of advancements, future iterations of the recipe recommendation module could contemplate the de novo creation of recipes using text or cooking graph representations, surpassing current NLP-based models. Furthermore, the optimal timing for dietary interventions, aimed at maximising radiotherapy outcomes, was beyond our current scope but warrants attention, potentially encompassing clinical trials assessing the interplay between dietary intervention timing and therapy outcomes. The current work, in general, provides a framework for the discussion of methodological approaches for the task of modulating radioresponse using bioactive molecules within foods. We consider this work as a first milestone approach in the design of genome-guided phytochemically-enriched recipes to improve RT outcomes in RC patients and envision its use as a baseline for future work. Our approach, centred on lipid metabolism modulation, offers a novel avenue to augment radiotherapy outcomes. Nevertheless, individual biological and health variations signify that it might not universally benefit all patients. Aspects like obesity and BMI, which intrinsically modify lipid metabolism and various physiological processes, could dictate the intervention’s effectiveness. In such instances, personalised strategies, ranging from dietary modifications to manage weight to pharmacological measures addressing obesity-related comorbidities, might be indispensable. By employing machine learning, our study enables recipe adjustments in line with identified potential radiotherapy modulators. This presents an opportunity for bespoke recipe alterations aligning with individual patient requirements, considering elements like obesity and BMI. Such a comprehensive, personalised treatment paradigm accentuates the essence of optimising radiotherapy outcomes and overall patient health. The flexibility of our approach also encompasses patient-specific data such as allergies, cost considerations, food preferences, and concurrent treatments, ensuring dietary compatibility and synergy.

Contrasting with gut microbiota modification strategies, our method prioritises direct dietary alterations aimed at cellular mechanisms, including lipid metabolism, rather than reshaping the gut microbiome. Nevertheless, dietary effects on gut microbiota composition and functionality are undeniable and can sway health outcomes, including therapy responses. Since the gut microbiota orchestrates the bioavailability of bioactive food molecules, these two strategies might be synergistically combined for a comprehensive therapeutic approach encompassing both cellular mechanisms and microbial interactions. Our proposed pipeline possesses the adaptability to address any disease given the knowledge of target genes, offering a holistic framework for recipe recommendations that complement prevailing treatment standards. We foresee evaluating these findings via clinical trials, providing participants with enriched recipes and evaluating dietary intervention impacts through outcomes such as progression-free survival (PFS) or disease-free survival (DFS). Moreover, the approach, although primarily centred on radiotherapy for rectal cancer, hints at the broader applicability, extending possibly to other therapeutic modalities or diseases.

## Conclusion

We introduce a network machine learning pipeline for predicting radioresponse modulators within foods and generating recipes to enhance RT response in RC patients. For the identification of radioresponse modulators within foods, we adopted a genomic-driven approach, hypothesising that these modulators should exhibit similar effects on protein networks as those observed in RT-resistant RC patients. To model the genomic effects of food molecules and phenotype, we integrated metric learning with biased RWR, mapping the influence of food molecules and the phenotype across a multiscale interactome. This process illuminated the proteins and biological functions most impacted. Overall, this study establishes a foundation for discussing methodological strategies aimed at modulating radioresponse through bioactive molecules in foods. We view this as a pioneering step in creating genome-guided, phytochemically-enriched recipes to enhance RT outcomes in RC patients and see its potential as a reference point for subsequent research.

## Methods

### Identifying radioresponse modulators

We propose the approach outlined in Fig. 1A for the identification of radioresponse modulators within foods. The core of our model is a graph $G=(\mathcal {V}, E)$ representing the multiscale interactome described by^23^, where nodes are proteins and biological functions, and edges represent protein-protein, protein-biological functions, and biological function-biological function interactions. Protein-protein interactions describe physical interactions between proteins. Protein-biological function interactions connect proteins to the biological functions they affect and biological function-biological function interactions represent the hierarchy of biological functions using the Gene Ontology’s Biological Processes^32^. For more details on the construction of the multiscale interactome, we refer the reader to^23^. Specifically, our graph *G* has $|\mathcal {V}| = N+M = 27,458$ nodes of which $N = 17,660$ are proteins and $M = 9798$ are biological functions. The phenotype, i.e., the over-expressed proteins in patients exhibiting RT resistance, is modeled as an *N*-dimensional vector ${\textbf {p}} \in \{0,1 \}^N$ where $p _i = 1$ if gene *i* is over-expressed and 0 otherwise. Similarly, protein targets of food molecules are represented as *N*-dimensional vectors ${\textbf {m}}^j \in \{0,1 \}^N$ where ${m}^j_i = 1$ if protein *i* is targeted by food molecule *j* and 0 otherwise. Information of 2100 food molecules and their targets are obtained from FoodDB^33^ and STITCH^34^ datasets. Using the multiscale interactome allows us to explain identified molecules, even when they seem unrelated to the phenotype. It additionally allows us to identify which biological functions are being modulated in cases where a short protein-protein path exists between food molecule targeted proteins and RC-resistant over-expressed genes, adding a level of interpretability.

#### Network propagation algorithm and metric learning

We combine a network propagation algorithm based on biased random walks with restarts with deep metric learning. The network propagation algorithm starts from initial nodes encoded in binary vectors encoding food molecules and the phenotype. At every step, the walker can restart its walk or jump to an adjacent node. The outputted diffusion profile measures how often each node in the multiscale interactome is visited by the RWR, encoding the effect of food molecules and the phenotype on every protein and biological function. In^23^, they optimise the edge weights of the algorithm for a multiscale-based drug-disease prediction task, in which an AUROC = 0.705 was achieved. The task involves predicting whether a drug treats a disease based on known drug-disease pairs taken from the Drug Repurposing Database, the Drug Repurposing Hub and the Drug Indication Database with only FDA-approved treatment relationships. Given that optimising the edge weights of the random walk algorithm has a very small effect on the prediction task (a fixed random walk probability of $\alpha = 0.64$ and edge-weights all being 1 gives 0.702 AUROC), we propose to fix the edge weights and optimise the weights of a multilayer perceptron (MLP) instead, using deep metric learning in order to minimise the distance between known drug-disease pair embeddings and maximise the distance to unknown drug-disease pairs. We set the propagation value of the RWR to 10 times the mean maximum propagated value over all drugs after propagating with $\alpha = 0$, giving a value of $\alpha = 0.64$. For each disease, we randomly sample both a positive drug (a drug which is known to be beneficial against the disease) and a negative drug (a drug which has no known benefit). This triple (disease, positive drug, negative drug) is passed to a MLP in order to get an embedding for the disease and the two drugs. A triplet loss is then used in order to minimise the distance between the disease and positive drug and maximise the distance to the negative drug. We use 5-fold cross-validation to optimize the model in the set of drugs and diseases (N = 1651), and use the trained model to give a ranking of food molecules based on distance to the phenotype embedding. In each split, we train the model for a maximum of 100 epochs using the Adam optimizer. Final propagation profiles reflect protein and biological functions affected. However, the model alone is not sufficient to filter out toxic molecules or metals from the food molecule database. Additionally, it is difficult for the model to learn whether the molecules affect the biological functions disrupted by the phenotype rather than directly targeting disease proteins or their regulators.

#### Filtering predictions

Using propagated profiles or the entity embeddings, we find the top 100 food molecules closest to the phenotype. These molecules affect similar proteins and biological functions as those responsible for radioresistance. Experimental evidence indicates that the phenotype-associated genes are over-expressed in patients exhibiting RT resistance leading to a positive modulation of lipid metabolism. Therefore, we use domain knowledge and literature search to filter out identified molecules with positive regulatory effects on lipid metabolism, leaving 33 modulators to retrieve the list of ingredients (Appendix A). Modulators belong to a myriad of compound classes including flavonoids, isoflavonoids, and bezenoids, in alignment with the current knowledge on chemotherapeutic bioactive molecules within foods^11^. Overall, predicted modulators are involved in cell signaling, cell growth and lipid metabolism. For example, Genistein works by inhibiting the Arachidonic Acid pathway, making it a suitable natural agent for cancer prevention and therapy^11^.

### Recipe optimisation module

Having found radioresponse modulators in the previous step, we propose to provide patients with recipes that maximize the number of ingredients with these molecules (Fig. 1C). Associations between foods and the molecules they contain are taken from FoodDB^35^, and a baseline set of recipes from the Recipe1M dataset^36^. Ingredients from these two datasets were preprocessed (turned to lowercase, spaces and plurals removed) and matched if they shared the first or last two words, or if they had the same word in the first or in the last position. This meant that ingredients such as king oyster mushroom and dried porcini mushroom were treated as being the same ingredient.

After combining these datasets, an enrichment score is calculated for each recipe based on the number of radioresponse modulators that they contain. Additionally, ingredient context embeddings from the BERT model^37^ are used to optimize the recipes and provide recommended ingredient substitutions to patients. These substitutions are done to increase the amount of anti-RT-resistance molecules whilst also preserving the recipe’s culinary attributes. Ingredient substitutions for the Recipe1M dataset were then found using the same method outlined in^38^. Starting with the bert-base-cased model in the Hugging Face library^39^, the BERT vocabulary was extended to include all the ingredients in the dataset. The BERT model, with a hidden representation of dimension 768, was then trained on the cooking instructions for each recipe in the dataset. Given that BERT gives different embeddings for the same ingredient in different contexts, there ends up being approximately 285,000 embeddings for all ingredients. For all the embeddings of a single ingredient, the 200 nearest neighbors were found using KNN and a substitute score given to other ingredients based on how often it appeared in the 200 nearest neighbors for all the embeddings. Suggested substitutes were then found for an ingredient by finding ingredients which had a score of over 100 and which were greater than 1/10 of the highest score for that ingredient.

To visualize the embedding space, we averaged all the embeddings for the same ingredient in order get a single embedding of dimension 768 for each ingredient. A 2D projection of this space using Principal Component Analysis is shown for a few of the ingredients in Fig. 4A. The suggested ingredient substitutes for a particular ingredient were then filtered to only include ingredients that had a higher number of molecules with potential for RT modulation than the initial ingredient. Some examples of these substitutions are shown in Fig. 4B. The number of beneficial molecules for each ingredient was found using the FoodDB database and is shown in Appendix A. Recipes in the Recipe1M dataset were optimized by looping through the ingredients and randomly selecting a substitute within the filtered list of substitutes. Additionally, it was constrained such that the same substitute can not be made for different ingredients within the recipe and a substitute suggestion which is already in the recipe is not allowed. Some examples of a mutated recipe are shown in Fig. 4C.

### Dietary recommendations

When recommending recipes to a patient, it is also important to take into account other factors such as allergies, food preferences and general nutritional guidance. The flexibility of our approach and scoring function makes this possible. We showcase this by further optimising our recipes to take into account allergies and food preferences. Additional input is given to the model in the form of a list of user allergies and a dictionary of user food preferences. The allergy list contains which of the 14 main food allergens the user has and the food preference dictionary has keys corresponding to ingredients and values being a score of 1–5 indicating the patient’s like of the food (1 indicating a strong dislike and 5 a strong like). In order to take into account this information, we create a database containing all the unique ingredients and whether they satisfy each of the 14 allergies. Given an allergy list input, we loop through all recipes and make an ingredient substitution for all ingredients where the patient is allergic. If there doesn’t exist a substitution or all substituted ingredients also cause allergies then the recipe is removed. We then optimise these new recipes as before to take into account both the number of radioresponse modulators and also the patient’s food preferences. This is done by making ingredient substitutions in a recipe if either the patient prefers the new ingredient or if there is an increase in the number of radioresponse modulators whilst also enforcing that there is not a reduction in the other.

### Supplementary Information

Supplementary Information.

## Data Availability

All data used in the paper is publicly available. Genome data can be collected from STRING^40^ (https://string-db.org), UniProt^41^ (https://www.uniprot.org), COSMIC^42^ (https://cancer.sanger.ac.uk/cosmic), and NCBI Gene^43^ (https://www.ncbi.nlm.nih.gov/gene/). Drug data can be extracted from DrugBank^44^ (https://www.drugbank.ca), DrugCentral^45^ (http://drugcentral.org), and STITCH^46^ (http://stitch.embl.de). Food data can be extracted from FooDB^47^ (https://foodb.ca) and STITCH^46^ (http://stitch.embl.de). The recipes can be obtained from Recipe1M^36^ (http://pic2recipe.csail.mit.edu/) and the Multiscale Interactome data and analysis from (github.com/snap-stanford/multiscale-interactome)^23^.
