# Supplementary material for: Genomic-driven nutritional interventions for radiotherapy-resistant rectal cancer patient
Source: Sci Rep. 2023 Sep 8;13:14862. doi: 10.1038/s41598-023-41833-8 (PMC10491580; doi:10.1038/s41598-023-41833-8)
Supplement: Supplementary file 1 — Supplementary Information. [file 41598_2023_41833_MOESM1_ESM.pdf]

Appendix

Appendix A. Table with predicted radioresponse modulators.

Name	ID	Annotation	Modulator/anticancer mechanism
Mangiferol	FDB005974	Benzopyrans	Mangiferin acts through a myriad of mechanisms to exert anti-inflammatory, immunomodulatory, cell cycle arrest, anti-proliferative, anti-apoptotic,anti-oxidative, anti-genotoxic, and anti-viral effects which cumulatively result in anti-tumour activity[?].
Dihydrosphingosine	FDB030824	Amine	inhibits prostate cancer progression by suppressing cell cycle and inducing cell apoptosis
Naphthalen-1-ol	FDB005841	Benzenoid	selectively toxic to human colorectal tumours[?].
Dichloromethane	FDB008159	Halomethane	Colorectal cancer chemoprevention via induction of apoptosis and G2/M cell cycle arrest mediated by cyclin A upregulation[?].
N,N-Dimethylformamide	FDB004724	Carboxylic acid derivative	can reversibly effect the reversion of cultured human colon carcinoma cells to less malignant cell types[?].
Cerebroside	FDB005463	Amine	the sphingoid base composition of cerebrosides reduce the cell viability by causing apoptosis in human colon cancer cells[?].
Levocarnitine	FDB000572	Quaternary ammonium salt	Carnitines slow down tumor development of colon cancer in the DMH-chemical carcinogenesis mouse model[?].
Bromelains	FDB001083	Diazine	inhibited CRC cell growth in cell lines and tumor growth in the zebrafish and xenograft mouse models. It also induced high levels of ROS and superoxide, plus autophagosome and lysosome formation.[?].
Quinoline	FDB011854	Alkaloid	The anti-colorectal cancer (CRC) activity of SL-1, a DNA-directed N-mustard-quinoline conjugate, was highly cytotoxic to various CRC cell lines. Experiments using xenograft models revealed that SL-1 was more potent than 5-fluorouracil (5-FU) and oxaliplatin for suppressing the growth of RKO and RKO-E6 (oxaliplatin-resistant subline) cells as well as metastatic SW620 cells[?].

alpha-Lipoic acid	FDB004339	Lipoic acids and derivatives	can effectively induce apoptosis in human colon cancer cells by a prooxidant mechanism that is initiated by an increased uptake of oxidizable substrates into mitochondria[?].
Thiocyanic acid	FDB013883	Thiocyanates	dietary isothiocyanates may exert tumor inhibitory effects, especially during earlier stages of the multistage process of carcinogenesis[?].
Lanthanum	FDB003776	Homogeneous lanthanide compounds	Dosage seems to be the pivotal factor for switching the biological effects from down- to up-regulation of cell growth; thus, low concentrations promoted cell survival and proliferation, but when concentrations increased, lanthanum exerted anti-proliferative and cytostatic/cytotoxic effects[?].
trans-resveratrol	FDB031212	Stilbene	Arachidonic Acid pathway inhibitory natural agent identified as cancer chemopreventive and therapeutic[?].
alpha-D-Galactose	FDB021787	Carbohydrate	induces necroptotic cell death in neuroblastoma cell lines[?]
NAD+	FDB031053	Nucleotide	A deregulation of the NAD+ levels has been associated with metabolic diseases and aging-related diseases, including neurodegeneration, defective immune responses, and cancer[?].
Syringic acid	FDB000514	Benzenoid	syringic acid-treated cells developed anti-cancer activities by losing MMP, cell viability, and enhancing intracellular ROS[?].
(R)-Sulforaphane	FDB012609	Sulfoxide	modulates many cancer-related events, including susceptibility to carcinogens, cell death, cell cycle, angiogenesis, invasion and metastasis[?].
beta-D-(1->3)-Glucan	FDB021471	beta-glucan	has a variety of effects on the immune system, including antitumor and anti-infective activities[?].
4-Phenylbutanoic acid	FDB022106	Benzenoid	inhibits cell proliferation, invasion and migration and induces apoptosis in glioma cells[?].
Kaempferol	FDB000633	Flavonoid	shown to be a potential chemotherapeutic agent to be used alone or in combination with 5-FU to overcome colon cancer drug resistance[?].
Naringin	FDB011866	Flavonoid	strong evidence for the association between treatment with naringin alone, or combined with other drugs and antitumor activity[?]

Genistein	FDB011828	Isoflavonoid	Arachidonic Acid pathway inhibitory natural agent identified as cancer chemopreventive and therapeutic[?].
Agmatine	FDB008310	Organonitrogen compound	had significant inhibitory effect on transplanted tumor growth in vivo and proliferation of tumor cells in vitro, and the mechanism might be a result of inducing decrease of intracellular polyamine contents[?].
D-Alanine	FDB022546	Carboxylic acid	expression of R. gracilis DAAO in tumor cells confers chemosensitivity to D-alanine that could be exploited as a novel cancer gene therapy paradigm[?].
Benzaldehyde	FDB014661	Benzenoid	shown antitumor activity against implanted Ehrlich carcinoma, adenocarcinoma and colon cancer[?]
Germacrone	FDB005347	Prenol lipid	exerts anti-cancer effects on gastric cancer through induction of cell arrest and promotion of apoptosis[?]
Celastrol	FDB022989	Prenol lipid	has been shown to suppress angiogenesis in colorectal cancer human cell lines[?].
Phytosphingosine	FDB023381	Amine	Phytosphingosine in combination with ionizing radiation enhances apoptotic cell death in radiation-resistant cancer cells through ROS-dependent and -independent AIF release[?].
Monodehydro-L-ascorbic acid	FDB029242	Furanone	may produce benefits in both prevention and treatment of cancer, by inhibiting malignant cell proliferation, and inducing differentiation and redifferentiation[?].
Ubiquinone Q2	FDB024036	Prenol lipid	Coenzyme Q10 or ubiquinone has been shown to have both anti-cancer and immune system enhancing properties when tested in animals[?].
Daidzein	FDB002608	Isoflavonoid	has been documented to have anti-carcinogenesis, anti-fibrotic, anti-diabetic, cholesterol-lowering and cardiovascular activity[?].
Hesperidin	FDB002680	Flavonoid	interacts with numerous recognized cellular targets and inhibits cancer cell proliferation by inducing apoptosis and cell cycle arrest[?].
Selenomethionine	FDB012370	Carboxylic acid	Selenium supplementation has been shown for many years to work as an anticarcinogenic agent both in epidemiology and in in vitro studies[?].

Table 1. Table with molecules within food with predicted RT response modulation activity

Appendix B. Highest Scoring Foods.

Name	Numer of RT modulators	RT modulators
Soy bean	5	Agmatine, Daidzein, Genistein, Kaempferol, Syringic acid
Fig	5	Benzaldehyde, Cerebroside, Kaempferol, Quinoline, Syringic acid
Black walnut	4	Benzaldehyde, Lanthanum, Naphthalen-1-ol, Syringic acid
Common bean	4	Benzaldehyde, Cerebroside, Daidzein, Genistein
Common pea	4	Daidzein, Genistein, Kaempferol, Syringic acid
Almond	3	Benzaldehyde, Kaempferol, Lanthanum
Blackcurrant	3	Benzaldehyde, Kaempferol, Syringic acid
Corn	3	Benzaldehyde, Genistein, Syringic acid
Lemon	3	Benzaldehyde, Hesperidin, Naringin
Mandarin orange	3	Benzaldehyde, Hesperidin, Naringin
Papaya	3	Benzaldehyde, beta-D-(1->3)-Glucan, N,N-Dimethylformamide
Pepper	3	Benzaldehyde, Hesperidin, Kaempferol
Sour cherry	3	Benzaldehyde, Genistein, Kaempferol
Sweet orange	3	Benzaldehyde, Hesperidin, Naringin
Tea	3	Benzaldehyde, Kaempferol, Quinoline
Pomegranate	3	Cerebroside, Kaempferol, Naringin
Broad bean	3	Daidzein, Genistein, Kaempferol
Mung bean	3	Daidzein, Genistein, Kaempferol
Common oregano	3	Kaempferol, Naringin, Syringic acid
Barley	2	Agmatine, Syringic acid
Broccoli	2	alpha-Lipoic acid, Kaempferol
Spinach	2	alpha-Lipoic acid, Kaempferol
Bilberry	2	Benzaldehyde, Syringic acid
Black elderberry	2	Benzaldehyde, Kaempferol
Cashew nut	2	Benzaldehyde, Lanthanum
Cloves	2	Benzaldehyde, Kaempferol
Common wheat	2	Benzaldehyde, Syringic acid
Garden tomato (var.)	2	Benzaldehyde, Kaempferol
Ginger	2	Benzaldehyde, Kaempferol
Green bell pepper	2	Benzaldehyde, Hesperidin
Hyssop	2	Benzaldehyde, Hesperidin
Kohlrabi	2	Benzaldehyde, Kaempferol
Loquat	2	Benzaldehyde, Kaempferol
Orange bell pepper	2	Benzaldehyde, Hesperidin
Parsley	2	Benzaldehyde, Kaempferol
Peach	2	Benzaldehyde, Kaempferol
Red bell pepper	2	Benzaldehyde, Hesperidin
Red raspberry	2	Benzaldehyde, Kaempferol
Spearmint	2	Benzaldehyde, Hesperidin
Sweet basil	2	Benzaldehyde, Kaempferol
Sweet bay	2	Benzaldehyde, Kaempferol
Yellow bell pepper	2	Benzaldehyde, Hesperidin
Adzuki bean	2	Daidzein, Genistein
Black-eyed pea	2	Daidzein, Genistein
Chickpea	2	Daidzein, Genistein
Lentils	2	Daidzein, Genistein
Lima bean	2	Daidzein, Genistein
Pigeon pea	2	Daidzein, Genistein
Scarlet bean	2	Daidzein, Genistein
Fenugreek	2	Genistein, Kaempferol

Common thyme	2	Germacrone, Kaempferol
Turmeric	2	Germacrone, Syringic acid
Cabbage	2	Kaempferol, Lanthanum
Carrot	2	Kaempferol, Syringic acid
Chicory	2	Kaempferol, Syringic acid
Cocoa bean	2	Kaempferol, Syringic acid
Common grape	2	Kaempferol, Syringic acid
Common hazelnut	2	Kaempferol, Lanthanum
Common persimmon	2	Kaempferol, Lanthanum
Common walnut	2	Kaempferol, Syringic acid
European plum	2	Kaempferol, Lanthanum
Evening primrose	2	Kaempferol, Syringic acid
Fennel	2	Kaempferol, Syringic acid
Garden onion	2	Kaempferol, Selenomethionine
Lettuce	2	Kaempferol, Lanthanum
Mango	2	Kaempferol, Mangiferol
Olive	2	Kaempferol, Syringic acid
Red beetroot	2	Kaempferol, Syringic acid
White cabbage	2	Kaempferol, Lanthanum
Wild carrot	2	Kaempferol, Syringic acid
Coconut	2	Lanthanum, Syringic acid

Table 2. Table with molecules within food with predicted RT response modulation activity

Appendix C. Dataset statistics.

As part of the pipeline introduced in this work, we use the pretrained model introduced by[?]. Here,[?]used a biased random walk with restarts algorithm across the multiscale interactome to compute profiles capturing perturbed proteins and biological functions as a result of drug or disease action. Perturbed profiles were used to predict drug-disease interactions (with drug-disease interactions representing effective treatments for diseases). The dataset used by the authors consisted of 1,661 drugs and their protein targets and 840 diseases and the proteins they disrupt (through effects like genomic alterations, altered expression, or post-translational modification), with 5,926 drug-disease interactions to predict. Using a 5-fold cross-validation strategy, weights of the biased random walk with restarts algorithm were optimized. In this work, we consider bioactive food molecules as potential 'treatments' for the phenotype or 'disease' encoded by the RT-resistant over-expressed genes, and use their optimized network propagation weights.

Drug and food molecule protein targets (Figure 1A) follow a somewhat similar distribution. We compute overlaps of protein targets within the drug dataset and between the food molecules and drug dataset: For each drug, we measure the percentage of protein target overlap between that drug and the rest of the drugs in the dataset, and do this for all drugs; for each food molecule, we measure the percentage of protein target overlap between that food molecule and the drugs in the dataset, and do this for all food molecules. Overlaps are defined as the fraction of drug (food molecule) protein targets that are also targeted by other drugs in the drug dataset. On average, 91% of drug targets are targeted by other drugs; and 57% of food molecule targets are targeted by other drugs.

Diseases in the multiscale dataset have a median of 9 associated affected proteins (Figure 1B), with the number of RT-resistant genes falling within the distribution. We compute overlaps of affected proteins within the disease dataset and between the phenotype and disease dataset: For each disease, we measure the percentage of affected protein overlap between that disease and the rest of the diseases in the dataset, and do this for all diseases; we compute the affected protein overlap between the phenotype and all diseases in the dataset. Overlaps are defined as the fraction of disease(phenotype)-associated genes that are also targeted by other diseases in the disease dataset. On average 94% of disease affected proteins are also associated to other diseases, and 58% of RT-resistant genes are also associated to other diseases in the dataset.

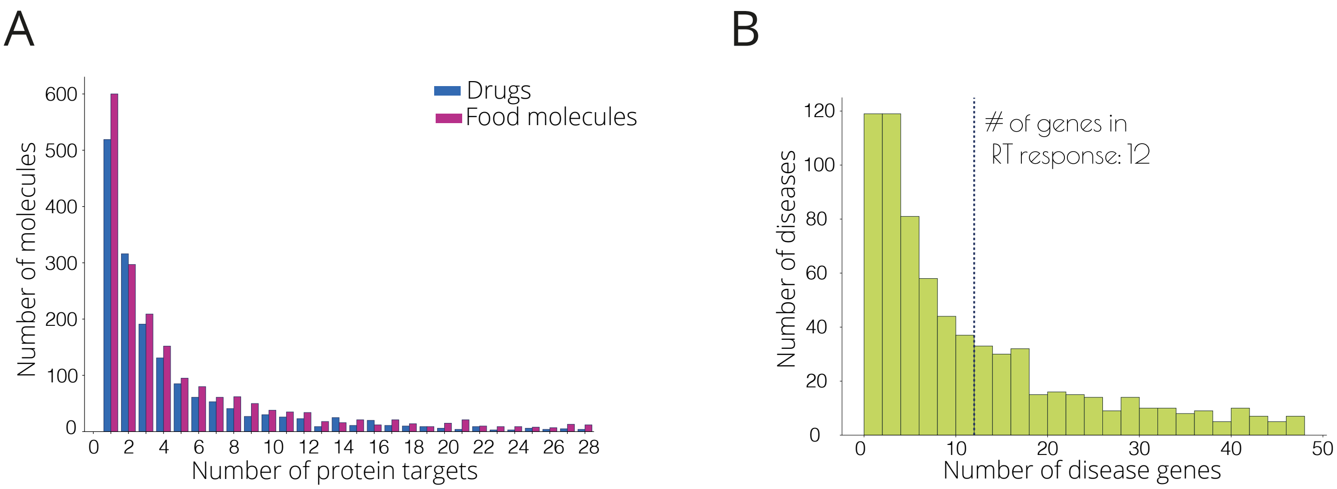

Figure 1. Distribution of drug and disease datasets used by pretrained model. **(A)** Distribution of drug and food molecule protein targets. Molecules with more than 30 protein targets are not plotted for clearer visualization (18 drugs, 167 food molecules) **(B)** Distribution of disease-associated genes. The median number of disease genes is 9. Diseases with more than 50 associated genes are not plotted for clearer visualization (129 diseases).
